# On the Capability of Artificial Neural Networks to Compensate Nonlinearities in Wavelength Sensing

**DOI:** 10.3390/s90402884

**Published:** 2009-04-21

**Authors:** Mohamed Lamine Hafiane, Zohir Dibi, Otto Manck

**Affiliations:** 1 Laboratoire d’Electronique Avancée, Département d’Electronique, Université de Batna, 05 avenue Chahid Boukhlouf 05000 Batna, Algeria; E-Mail:zohirdibi@yahoo.fr (Z.D.); 2 Institut für Technische Informatik und Mikroelektronik, Technische Universität Berlin, Germany

**Keywords:** Buried photo *PN* junctions, Artificial Neural Network, wavelength measurement

## Abstract

An intelligent sensor for light wavelength readout, suitable for visible range optical applications, has been developed. Using buried triple photo-junction as basic pixel sensing element in combination with artificial neural network (ANN), the wavelength readout with a full-scale error of less than 1.5% over the range of 400 to 780 nm can be achieved. Through this work, the applicability of the ANN approach in optical sensing is investigated and compared with conventional methods, and a good compromise between accuracy and the possibility for on-chip implementation was thus found. Indeed, this technique can serve different purposes and may replace conventional methods.

## 1. Introduction

The use of wavelength measurement has a wide range of applications, varying from fiber-optic communication to biological purposes, such as DNA sequencing, including many engineering applications. This increase of applications has provided motivation to improve all elements of the optical sensing chain, as well as the photodetector fabrication process, conditioning circuits and readout algorithms. In this sense, the most state-of-the-art BICMOS (combination of bipolar and CMOS technology) optical sensors involve a trade-off between implementation cost and readout accuracy.

**Figure 1.**
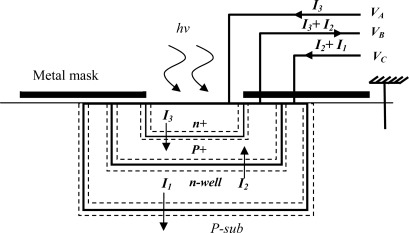
Cross-section view of BJT.

**Figure 2.**
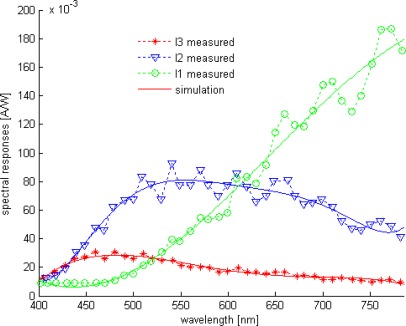
BTJ Spectral responses [1].

In general, the well known methods for either color identification or wavelength measurement use color filters. In principle three photodetectors are covered respectively by red, green and blue filters which increases both silicon surface and implementation cost due to the deposition of optical filters (nonstandard BICMOS processing) [1–3]. In this perspective, the buried triple *pn* junctions (BTJ) structure, using BICMOS process (Figure 1), provides a promising alternative. Unlike the conventional photodetectors the BTJ has three outputs according to captured light; hence three different spectral responses (Figure 2) carry the wavelength value. Different process parameters, such as doping profiles allow conceiving three bandpass filters curves adjusted, with a limited resolution, in blue, green and red areas [1]. Due to process parameters variations from one chip to another, the bandpass filters shape change significantly, and as a result, this lowers the readout accuracy. This drawback can be compensated using learning algorithms, such as artificial neural networks.

In the past few years, artificial neural networks (ANNs) have emerged in many engineering applications as a learning technique to achieve complex tasks, as well as image analysis, high nonlinear modeling and system control [4,5]. They present interesting characteristics, such as the capability of universal approximation, generalization, and fault tolerance [6]. Furthermore, it is shown that ANNs based approximation of measurement data perform better than those of classical methods of data interpolation, in particular the mean square regression [7]. Thus, ANNs are commonly used for measurement sensor systems, in this scope, several works has been reported in [8–20], where the aims of their applications are to increase the selectivity, sensitivity, and reliability of many sensor types. This work carries this ideas one step further by applying similar techniques for wavelength readout, structured in a row of BTJs, in purpose of an embedded system for real time applications; featuring relative low full-scale error and a compatibility with BICMOS process which increase the system portability.

## 2. Modeling and Problem Formulation

The basic structure of the CMOS BTJ is illustrated in Figure 1. Three buried junctions are stacked between p-subtract to n+ diffusion, thus the device has three outputs through contacts in the peripheral areas: p+ diffusion, n+ diffusion and n-well. All junctions operate in reverse bias mode by applying external voltages V_A_, V_B_ and V_C_ (with V_B_ < 0, V_A_ > V_C_ > 0). In principle, the absorption of visible light in the silicon bulk induces generation of electron-hole pairs; where the generation rate depends on the wavelength of the incident light and on the depth from the silicon surface. Therefore, three stacked junctions result different spectral responses depending on the junction depth [1,21,22]. Figure 2 shows an example of BJT spectral response given at room temperature, the characterized cell is fabricated using 1.2 μm standard BICMOS process with an area of 28 by 28 μm [1]. The spectral response curves are approximated with fifth degree polynomials (1), with a limited precision.

(1) $$I_{n}=\sum_{i=0}^{5}a_{\mathrm{in}}.\lambda^{i}$$

where, *λ* is the wavelength and *In* is the photocurrent of the three junctions. This analytical approach can be used to get a linear transformation between the light wavelength and the currents measurement. In this case, the photocurrent variation versus light power and temperature is assumed linear.

Obviously, the device can detect either light intensity or wavelength variation. Indeed, the resulting currents are proportional to both variations, while the photocurrent ratio is sensitive to the optical wavelength [23]. The use of photocurrents ratios (I_1_/I_2_ and I_1_/I_3_) eliminate the need to fit BTJ spectral response to bandpass curves of optical RGB filters (red, green and blue), which is more suitable for colors recognition. Depending on photocurrent ratios, the wavelength can be modeled as non-linear function (2) of both ratios *I_1_/I_2_*, *I_1_/I_3_* and the temperature *T.*

(2) $$\lambda =f^{-1}\left(\frac{I_{1}}{I_{2}},\frac{I_{1}}{I_{3}},T\right)$$

**Figure 3.**
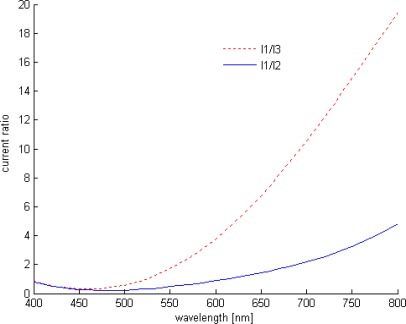
Photocurrent ratios *vs.* wavelength (simulation).

This asymmetric response is illustrated further in Figure 3, which shows simulated current ratios variation as function of wavelength. This model provides sufficient accuracy in determining the wavelength, including temperature influence on sensor response characteristics. However, the device response is 3D nonlinear function which gives rise to several difficulties for on-chip readout. Either an analytical or a numerical model can be used for wavelength readout; their drawbacks are the readout error caused by analytical model approximations and the time cost induced by numerical model calculations. Therefore, ANNs present an interesting alternative, where the network is trained to invert the sensor's transfer function “ *f^−1^*” by feeding current ratios *I_1_/I_2_*, *I_1_/I_3_* and the temperature *T*.

## 3. ANN Based-on Signal Readout

**Figure 4.**
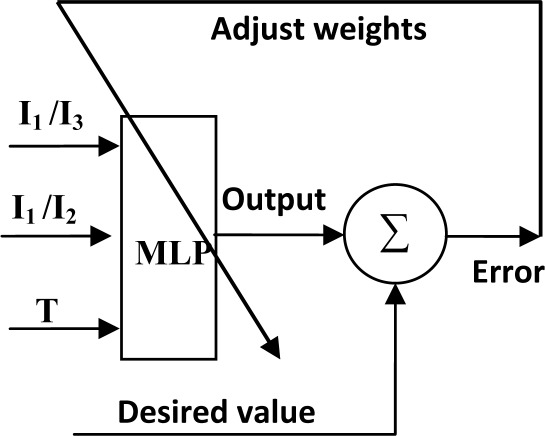
MLP-based wavelength readout (training set).

ANNs are powerful data modeling tools, where the advantage lays in their ability to represent both linear and non-linear models by learning directly from data measurements. In this field, the multilayer perceptron (MLP) is the most used ANN concept, according to the well known ANN state-of-the-art. It’s demonstrated in [24,25] that a MLP with one hidden layer suffices to approximate any function with an arbitrary precision (universal approximation theorem). MLP is a supervised network, where the training data consists of inputs and desired outputs. The error between MLP outputs and desired outputs is used to update the network weights (Figure 4), using back propagation (BP) algorithms [6]. In this scope, the magnitude of the problem is often seen from two perspectives: examples number necessary to attain a good convergence and the network size.

Based on measurement values, input/output dataset vectors, arranged as: *X⃗* = [*I*1/*I*2, *I*1/*I*3, *T*,*λ*], are used for the MLP training phase with 234 samples, and tested in a separated set with 36 samples. Once the training set is achieved by reaching the minimum mean square error (MSE), of the estimated wavelength, the network performance is checked again using test samples. This procedure is applied to several networks having one hidden layer and different neuron numbers per layer.

**Figure 5.**
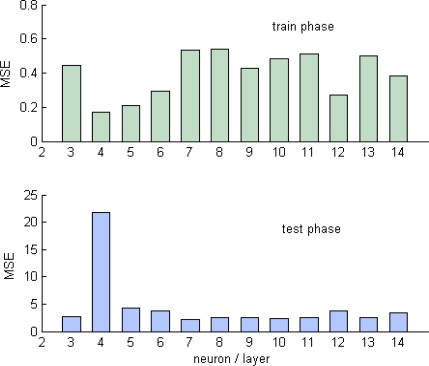
MSE of test and training for different architectures.

For these different architectures, both train and test MSE is evaluated and compared, the results are shown in Figure 5. Starting from 3 neurons per layer up to 14 neurons, the most training errors are less than 0.8, while the minimum test error is attained with 7 neurons per layer.

**Figure 6.**
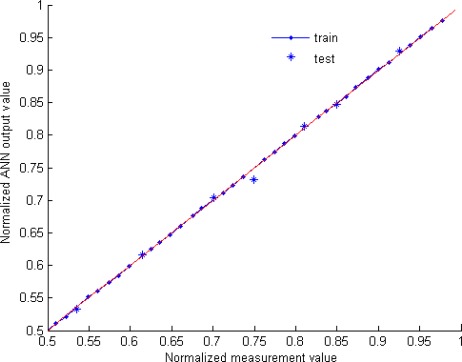
ANN model validation.

For this structure the MSE test is equal to 2.2 which represents a full scale error less than 1.5%. Thus, the selected network has one hidden layer containing seven neurons. The ANN optimized parameters are summarized in Table 1. Furthermore, Figure 6 illustrates the predictive property of the optimized network, the ANN response and measurement values are compared, thus a good agreement between measurement and ANN model is founded. It’s noted that the full scale error (%FS) is expressed as a percentage of the ratio between the absolute error and the maximum output (wavelength) variation range.

## 4. Implementation and Simulation Results

**Figure 7.**
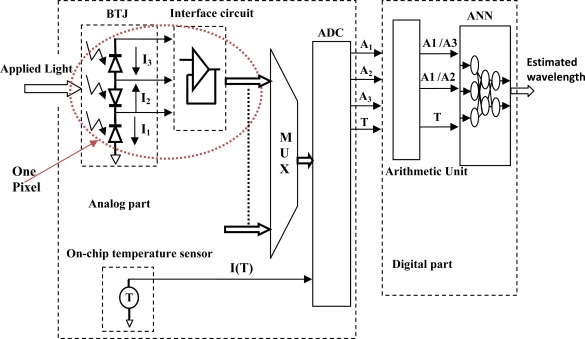
Top level simulation diagram.

The resulting currents of one BTJ cannot be directly exploited; therefore a typical acquisition chain is employed. One pixel path contains an analog interface circuit for BTJ conditioning, analog multiplexer, analog to digital converter (ADC), on chip temperature sensor and digital part for logical and mathematical calculations including ANN model (either on-chip FPGA or CPU implementation is possible). The complete embedded system top level diagram is shown in Figure 7. For a typical measurement, the analog interface circuit adapts BTJ signal to ADC voltage input range (both interface circuit and BTJ form one pixel) while the analog multiplexer allows the selection of desired pixel signal. Once the A/N conversion is achieved, the ANN data inputs *A_1_*/*A_2_*, *A_1_*/*A_3_* and *T* according to *I_1_/I_2_*, *I_1_/I_3_* and *I*(*T*), are calculated and fed to the net, thus the ANN model yields the estimated wavelength.

**Figure 8.**
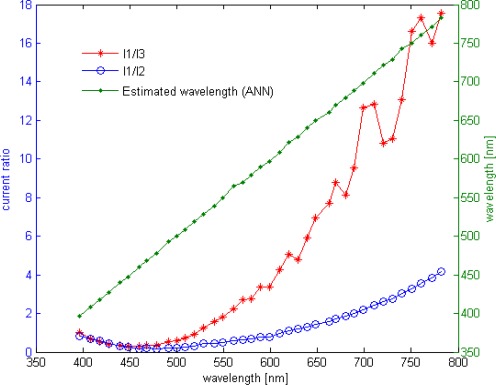
Estimated wavelength, I_1_/I_2_ and I_1_/I_3_ *vs.* applied wavelength.

**Figure 9.**
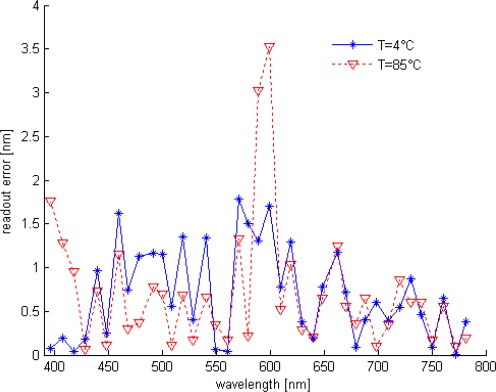
Readout error *vs.* wavelength at T = 4 and 85 °C.

**Figure 10.**
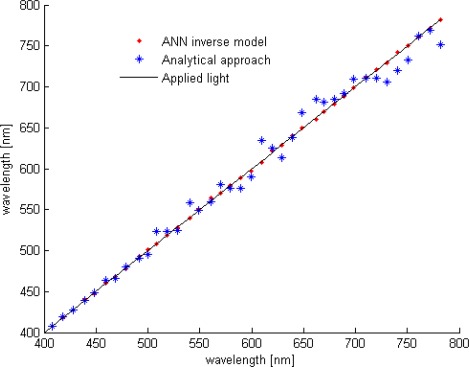
Comparison of ANN and analytical approach.

**Figure 11.**
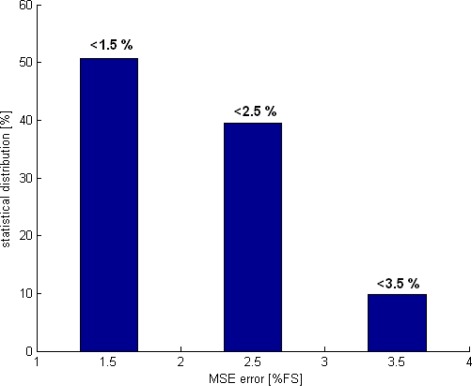
Statistical MSE distribution.

The wavelength row sensor performances is tested and evaluated with Cadence post simulation tools, based on the above diagram and high accurate BTJ model. For one pixel path, a row of different wavelength lights is applied starting from 400 up to 780 nm. At room temperature, the estimated wavelength and both current ratios are plotted in Figure 8, while Figure 9 shows the readout error versus wavelength at temperatures of 4 and 85 °C. According to these results, the smart sensor response presents a good linearity and a full scale error less than 1.5% over the temperature range of 80 °C. The ANN based-on wavelength readout is compared with analytical approach, previously explained, both responses are reported in Figure 10. Furthermore, Due to the non-ideal component characteristics, such as mismatch and tolerance, a statistical study to predict system reliability is done, using statistical models for BTJs and pixel path devices, the ANN approach performance is evaluated once more. The statistical distribution according to the mean square error (MSE) is reported in Figure 11. The obtained results shows that, in 50.7% of the tested cases the error yielded is less than 1.5%.

## 5. Conclusions

The use of an artificial neural network approach to achieve wavelength readout is promising. Indeed, the ANN can learn the BTJ sensor properties, and thus they can produce the inverse model, which is used as readout interface to improve sensor performances. Both ANNs model and BTJs can be implemented in one chip, using standard BICMOS process, featuring a good agreement between obtained performances and the implementation cost. The challenge of such application is how to keep a good chip-yield when the chip is in mass production; in fact, devices mismatch and tolerance, which refers to the used technology, decrease the system performances. Depending on both targets the maximum error and the chip-yield, the ANN weights adaptation for each pixel is needed. However the required space memory for such weights storage is relatively low compared to an on-chips VLSI memory capacitance. Furthermore, increasing applications of artificial neural networks carries the motivation for intensive research in this field. Thus dramatic improvements are yielded every year; such as, ANNs on-chip learning techniques. This topic provides good perspectives for the present work.

## Figures and Tables

**Table 1.** ANN optimized parameters.

**Parameters**	**Optimized values**	
Architecture	Normal feed-forward MLP	
Hidden layer	1	
Training algorithm	Back-propagation	
Number of neurons	Input layer	3
	Hidden layer	7
	Output layer	1
Transfer function	Hidden layer	Sigmoid
	Output layer	Linear
Output range	Wavelength (nm)	
	Max	780
	Min	400
Data base size	Training set	234
	Test set	36
